# Colorado State Medical Association

**Published:** 1895-08

**Authors:** 


At the recent meeting of the Colorado State Medical Association at Denver, two papers on castration were read. Dr. J. M. Blaine, well known to the Texas profession, opened the discussion. He said,—as doctors saw more of this thing of sexual perversion and realized more fully than others the necessity for castration, he thought they should "start the ball in motion." Here his voice was drowned by a torrent of applause, which lasted till his five minutes expired, and he was thus cut off from an opportunity of finishing his remarks. He claims it was unintentional, but many say it was a deliberate diabolism, especially as about fifteen lady physicians were present.

